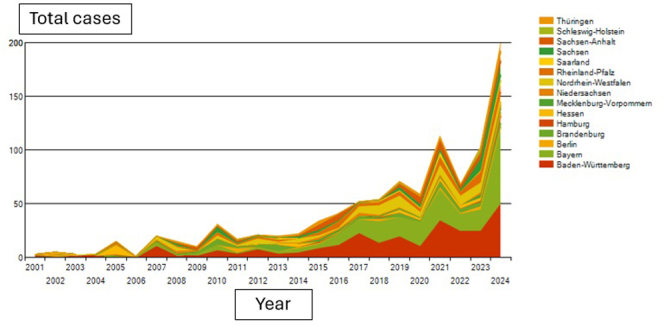# Tularemia With Necrotizing Mediastinal Lymph Nodes

**DOI:** 10.1016/j.atssr.2025.11.022

**Published:** 2025-12-17

**Authors:** Matthias Grott, Hauke Winter, Lisa Mager, Elvira Stacher-Priehse, Rudolf Hatz, Johannes Haag

**Affiliations:** 1 Department of Thoracic Surgery, Heidelberg University Hospital, Heidelberg, Germany; 2 Translational Lung Research Center Heidelberg (TLRC-H), Member of the German Center for Lung Research (DZL), Heidelberg, Germany; 3 Praxis Dres. Mager, General Practitioners, Neubeuern, Germany; 4 Department of Pathology, Asklepios Pulmonary Hospital, Gauting, Germany; 5 Center for Thoracic Surgery Munich, Ludwig-Maximilians-University of Munich (LMU), Munich, and Asklepios Medical Center, Member of the German Center for Lung Research (DZL), Asklepios Pulmonary Hospital, Gauting, Germany

## Abstract

Tularemia, also known as rabbit fever, is a rare zoonotic infectious disease transmitted by air, arthropods, food, and water caused by bacteria of the genus *Francisella.* Because of its multiple transmission routes and low infectious doses, *Francisella tularensis* is listed as a biological weapon. In rare cases, tularemia can lead to pneumonia and necrotizing mediastinal and hilar lymph nodes. Herein we present the case of a 43-year-old female patient with an isolated mediastinal mass, initially thought to be a solid tumor. Further investigations led to the diagnosis of tularemia. The patient’s medical history, treatment, and follow-up care are described.

Tularemia, commonly known as rabbit fever, is caused by the gram-negative, facultative intracellular, aerobic coccobacillus *Francisella tularensis*.^1^^,^^2^ The 2 subspecies *Francisella tularensis tularensis* (type A, Northern America only, more severe) and *Francisella tularensis holarctica* (type B, Northern Hemisphere) cause disease in humans.^2^ Routes for human transmission are arthropod borne (eg, tick bites), airborne, foodborne, and waterborne.^2^ *F*. *tularensis*–infected carcasses of rodents are most likely the source of bacterial aerosols or contaminated water. Inhalation of infective aerosols (eg, during farming activities: contact with infected hay, disposal of dead animals) may cause respiratory tularemia.^1^ Human-to-human transmission is known to occur only in solid organ transplants.^3^ The incubation period is between 3 and 21 days.^1^^,^^2^ The clinical presentation is divided into 6 different patterns^1^^,^^2^: ulceroglandular (lymphadenopathy, skin ulcerations), glandular (lymphadenopathy without skin lesions), oculoglandular (conjunctivitis, preauricular lymph nodes), oropharyngeal (tonsillitis, cervical lymphadenopathy), typhoidal (persistent fever, abdominal discomfort), and pneumonic (pneumonia, thoracic lymphadenopathy). Bacterial culture from recovered viable bacteria (blood, biopsy specimens, swabs, sputum, abscess aspirates) is the “gold standard” in tularemia diagnosis. Results of serology (IgG, IgM) are positive 10 to 20 days after infection.^1^^,^^2^ Multiple polymerase chain reaction (PCR) assays for molecular detection in recovered specimens are available. Minimum biosafety level 2 (level 3 for research) is recommended for (special) diagnostic laboratories handling *F*. *tularensis* materials.^1^ Standard treatment of adults is 14 to 21 days of orally administered ciprofloxacin or doxycycline (in combination with gentamicin in severe cases).^1^^,^^2^ The mortality rate is <1% for type B, 2% to 3% for type A, and up to 60% in patients who have been treated improperly. Reporting to national control registries is mandatory.^2^ Because of its multiple transmission routes and low infectious doses (10 to 15 bacterial cells), *F*. *tularensis* is listed as a biological agent for bioterrorism by the World Health Organization.^1^^,^^4^

The patient is a 43-year-old White woman with multiple sclerosis and implanted pacemaker for congenital third-degree atrioventricular block. She has a history of smoking (2.5 pack-years) and no long-term medication. Family history was positive for colon cancer (father, paternal grandfather). She presented to her general practitioner in rural southern Germany with diffuse gastrointestinal discomfort, diarrhea, and nausea. Besides a mildly distended abdomen, the physical examination did not reveal any abnormalities. The abdominal ultrasound findings and laboratory values were normal, so no specific therapy was initiated. Four weeks later, the patient returned with fever up to 40° C and a cough. Laboratory results showed a slightly elevated C-reactive protein level (47.4 mg/L) and neutrophilia as well as leukocyturia and microhematuria. The patient was given fosfomycin for a urinary tract infection. This initially led to clinical improvement. However, at the scheduled follow-up examination 1 week later, she suffered from severe shortness of breath and significantly reduced physical performance. There was no cough, hemoptysis, expectoration, or lymphadenopathy. Auscultation revealed normal breath sounds. After an unremarkable cardiac ultrasound examination, computed tomography with contrast enhancement was performed to rule out pulmonary embolism. The scan showed a 5-cm mass and enlarged mediastinal lymph nodes (stations 4L and 5/6). There was no radiologic evidence of pulmonary artery embolism (Figure 1; Video 1). Because of suspicion of a malignant tumor, the patient was immediately referred to the pulmonology department of the local hospital for bronchoscopic biopsy of the tumor. Histopathologic examination revealed normal lymphoid tissue with no signs of granulomas or malignancy. Therefore, a left-sided triportal video-assisted thoracoscopic biopsy was performed in the surgical department of the local hospital. Histopathologic examination revealed normal thymus tissue and perithymic fatty tissue. Because of the discrepancy between the histologic result and the radiologic findings, the patient was referred to a tertiary referral center for a thoracic surgeon to perform another left-sided uniportal video-assisted thoracoscopic biopsy. Owing to adhesions of the left upper lobe to the mediastinum, a wedge resection of the left upper lobe was performed. The mediastinal lesion was biopsied. The frozen section examination revealed suspected lymph node tuberculosis. Therefore, antitubercular antibiotic therapy was started immediately after surgery and switched to levofloxacin because of a negative PCR and interferon-γ release assay after 48 hours. Antibiotic therapy was continued on the basis of preliminary histopathologic examination, which revealed a granulomatous lymph node abscess but no signs of acid-fast bacilli (Figure 2). A biopsy specimen and blood sample from the patient were sent to the Institute for Microbiology of the German Armed Forces for referral testing and serologic examinations (toxoplasmosis, brucellosis, leptospirosis, Q fever/*Coxiella burnetii*, *Bartonella henselae*, and tularemia). PCR testing of the sample was positive for DNA from *F*. *tularensis holarctica*. Serology showed elevated IgG and IgM antibodies to *F*. *tularensis* in the enzyme-linked immunosorbent assay (IgG >300 U/mL [normal, <10 U/mL]; IgM, 72 U/mL [normal, <10 U/mL]). Oral antibiotic therapy was switched from levofloxacin to ciprofloxacin (total of 14 days of fluoroquinolone). This led to immediate relief of her symptoms. Follow-up scans 4 weeks (imaging not shown) and 6 months after treatment (Figure 3; Video 2) showed no remnants of the disease. The patient is in excellent health.

**Figure 1:**
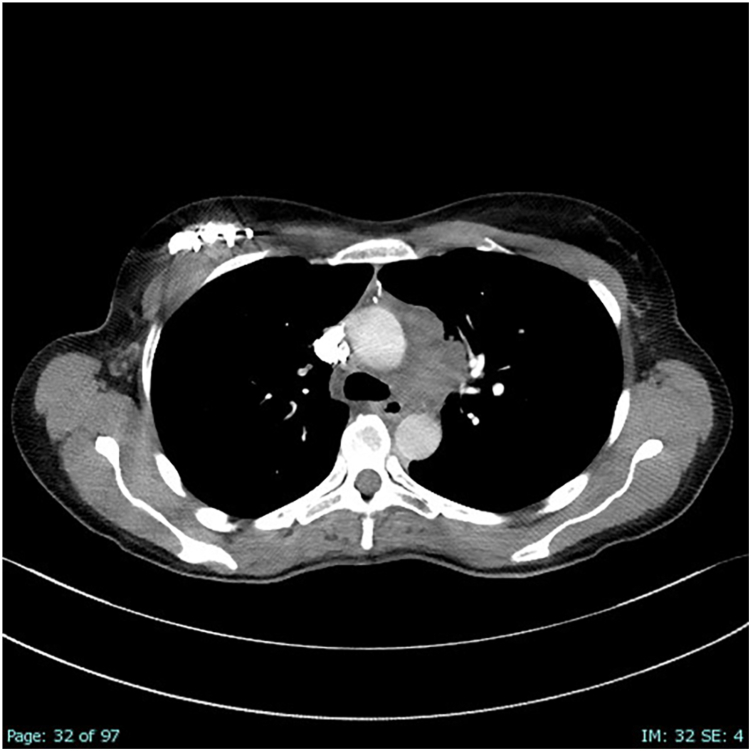
Initial computed tomography scan of the thorax showing the left-sided mediastinal mass.

**Figure 2:**
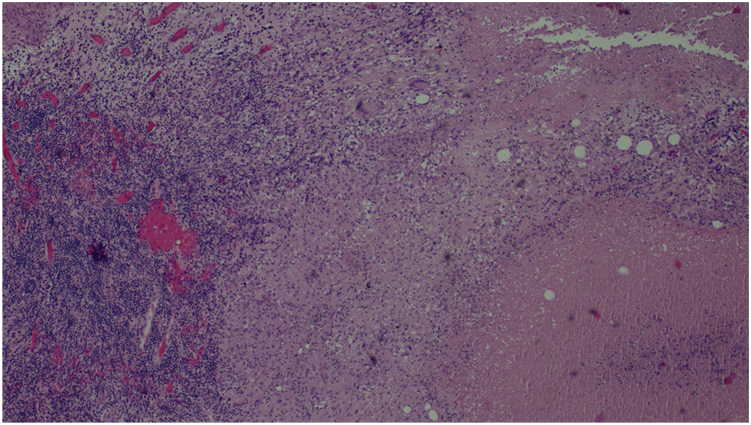
Extensive geographic necrosis with scant neutrophilic granulocytes and demarcated by palisading histiocytes, epithelioid cells, macrophages, and lymphocytes.

**Figure 3:**
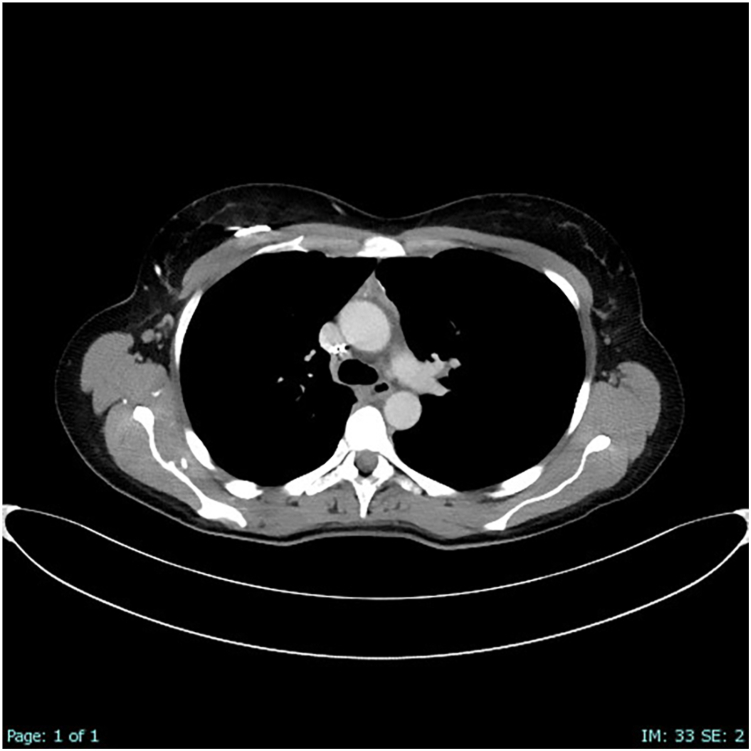
Computed tomography scan of the thorax 6 months after treatment.

## Comment

In Germany, *F*. *tularensis holarctica* is the only subspecies that causes tularemia in humans, with incidence increasing slightly during the past 2 decades (Supplemental Figure).^5^^,^^6^ Global warming with increased activity and greater distribution of vectors/arthropod-borne infections, better laboratory and serologic tests, improved national reporting systems, and increased contact between humans and animals could be responsible for the increase in tularemia infections.^2^^,^^5^ We were unable to trace the route of infection in our patient. The case presents a good example of the need for close interdisciplinary collaboration and expanded diagnostics. This is particularly important when tularemia mimics malignant disease in radiologic imaging.^7^ “When you hear hoofbeats, think of horses, not zebras”—in our case, maybe think of unicorns.

## Figures and Tables

**Supplemental Figure:**