# Supplementary material for: Tularemia With Necrotizing Mediastinal Lymph Nodes
Source: Ann Thorac Surg Short Rep. 2025 Dec 17;4(2):513–5. doi: 10.1016/j.atssr.2025.11.022 (PMC13245286; doi:10.1016/j.atssr.2025.11.022)
Supplement: Supplemental Material [file mmc1.docx]

# Supplemental Material

**Supplemental Figure 1**

Tularemia cases in Germany over the last 20 years with rising incidence stratified by 16 federal states (source: Robert Koch Institut, modified for English).
